# Preventive Behavioral Insights for Emerging Adults: A Survey during the COVID-19 Pandemic

**DOI:** 10.3390/ijerph18052569

**Published:** 2021-03-04

**Authors:** Sunhee Park, Beomsoo Kim, Kyoung A. Kim

**Affiliations:** 1 Barun ICT Research Center, Yonsei University, 50 Yonsei-ro, Seodaemun-Gu, Seoul 03722, Korea; sunny372@hanmail.net; 2 Graduate School of Information, Yonsei University, 50 Yonsei-ro, Seodaemun-Gu, Seoul 03722, Korea; beomsoo@yonsei.ac.kr; 3 Department of Nursing, Yeoju Institute of Technology 338, Sejong-ro, Yeoju-si 12652, Korea

**Keywords:** preventive behavior, emerging adulthood, COVID-19, infodemic, subjective norm

## Abstract

Emerging adulthood is an important period for establishing health behavior patterns in life. This study aimed to examine factors related to preventive behaviors of emerging adults during the COVID-19 pandemic. A descriptive online survey design was used. Data were collected using a self-administrated, 28-item questionnaire completed by 239 undergraduate students from a university in Seoul, South Korea. The questionnaire was developed based on previous studies and the guidelines of the World Health Organization about COVID-19 preventive behaviors. The mean age of participants was 21.97 years, and the average score for COVID-19 preventive behaviors was 4.13 (SD: ±0.42) on a 5-point scale. Hierarchical regression analyses revealed that subjective norms related to parents (β = 0.425, *p* < 0.001), issue involvement related to COVID-19 (β = 0.160, *p* = 0.024), and sex (β = 0.137, *p* = 0.029) were significant factors related to preventive behaviors of emerging adults after controlling for demographic characteristics. The variables explained 20.1% of the variance in preventive behaviors. The results of this study suggest that better strategies for subjective norms related to parents and issue involvement related to COVID-19 must be considered to improve emerging adults’ preventive behaviors.

## 1. Introduction

The novel coronavirus pandemic (hereinafter COVID-19) is a highly contagious respiratory disease that has spread to more than 120 countries globally after the first infection was confirmed in Wuhan, China, on 1 December 2019. In Korea, the first COVID-19 case was confirmed on 20 January 2020, and as of 20 January 2021, the number of cases has reached almost 80,000 [1].

Apart from vaccines, which are becoming available, there are no specific treatments for COVID-19 to date. Reducing the transmission of the virus is the most effective way to control the pandemic, requiring rapid and widespread behavioral changes that include these three measures: compliance with personal hygiene rules, social distancing, and disease management [2]. Compliance with personal hygiene includes frequent hand washing; appropriate hand cleaning techniques, such as washing with soap and water for at least 30 s and using alcohol-based hand sanitizer; and regularly disinfecting the surfaces of mobile phones [3,4]. Further, people are advised to wear disposable face masks; to cough or sneeze into their arm or elbow (instead of their hands); and to avoid touching their eyes, nose, or mouth. Social distancing techniques include staying at least six feet away from other people when in public, avoiding crowds or crowded places, refraining from using public transportation, and following work and quarantine restrictions [5]. Further behavioral changes require testing for the virus and monitoring contacts. People are asked to proceed with testing if they suspect they have symptoms or have been in contact with someone infected and to allow their contacts to be monitored [1].

The “infodemic” is another serious issue related to the increasing use of social media and communication technologies [6]. Infodemic refers to the spread of false information as fast as—or even faster than—the disease itself, and prevention is essential in pandemic situations [7]. In order to minimize COVID-19 transmission, it is necessary to convey accurate and comprehensible information to the public.

To aid in disease management, innovative solutions such as artificial intelligence (AI), machine learning and deep learning, nanomedicine, novel technologies for vaccine development and therapeutics, novel mathematical modeling, big data, the internet of things (IoT), telemedicine, robots, and 3D printing technology are imperative due to limited resources and the immediate requirements for medical supplies, healthcare support, and treatments [8]. Although these advanced technologies help to diagnose, test, and manage new infectious diseases, compliance with preventive behaviors at the individual level is still the best measure to prevent infections worldwide. 

Psychological responses and subjective norms have been observed to be related to individual protective behaviors in previous global pandemics [9,10,11,12]. Risk perception, such as the perception of susceptibility and the severity of a disease, is significantly related to individual protective behaviors during the COVID-19 situation and other pandemics [13,14]. Subjective norms are individuals’ behavioral norms or beliefs that are shaped by their social environment such as friends, parents, or significant others; these social-environmental factors act as motivation for conforming to these norms [15]. Several studies have sought to demonstrate the effect of subjective norms in terms of health behaviors [16,17,18,19]. Recent evidence suggests that social norms, specifically among friends and family, are likely to lead people to adopt more preventive behaviors and to perform these behaviors more frequently during the COVID-19 pandemic in the USA [20]. Issue involvement refers to how personally relevant individuals find a specific issue [21], and it has been found to be related to individuals’ health behaviors [22]. Therefore, during a new pandemic, higher issue involvement increases the likelihood of adhering to required health behaviors by increasing the requirement to care for one’s health and pursue accurate and logical health information [23]. 

Based on recent studies, being older, female, and more educated is associated with a higher chance of adopting the preventive rules [2]. In a study conducted during the COVID-19 pandemic, it was shown that sex (female), knowledge, personal health status, perceived severity, and social support were factors related to preventive behaviors in Korea [24].

Emerging adulthood has been identified as a new developmental phase, which is characterized by a period of exploration for people 18–29 years old before they settle into established adult roles [25]. During the emerging adulthood phase, individuals expand their relationship network, establish mature relationships with peers, and develop closer relationships with their families and siblings. This has been shown to be the healthiest period, physically, in an individual’s life span and is an important age for establishing long-term health behavior patterns [26]. It is common for emerging adults to be exposed to harmful environments and detrimental health cultures after graduating from high school; therefore, appropriate management and preventive measures are crucial during this period [27].

Emerging adults most often experience less severe symptoms but still contribute to the spread of the virus. Within the context of the COVID-19 pandemic, it is crucial to understand the psychological factors of preventive behaviors to manage infections [11]. This study aims to assess the preventive behaviors and to examine the factors related to preventive behaviors among emerging adults as psychological responses and subjective norm variables within the context of the COVID-19 pandemic. 

## 2. Methods

### 2.1. Instrument Development

An online, 28-item questionnaire was developed based on previous studies [16,18,28,29] and the COVID-19 prevention guidebook provided by the WHO [30] and the Korea Centers for Disease Control and Prevention [8]. A group of eight experts was invited to assess the content validity of the questionnaire. They reviewed the draft questionnaire and provided insightful comments and suggestions regarding the same. In addition, an item-level content validity index (I-CVI) and a scale content validity index (S-CVI/Ave) were used to analyze the questionnaire. The questionnaire was found to have good content validity (S-CVI/Ave = 0.79). It incorporated six types of preventive behaviors with 10 items. Such behaviors included (1) hand hygiene (frequency and methods), (2) mobile phone hygiene, (3) wearing masks (frequency, method, and not touching the surface), (4) coughing etiquette, (5) social distancing and avoiding public gatherings, and (6) infodemic behaviors (producing and sharing). The questionnaire comprised 3 items regarding issue involvement, 5 items on risk perception, and 10 items on subjective norms related to parents, friends, school, non-official sources, and official sources (supplementary file).

### 2.2. Participants and Procedure

The participants were undergraduate students from one university in Seoul, South Korea. The inclusion criteria for the participants were that they be between 18 and 29 years old and that they consented to participate. The necessary sample size was determined to be 194 participants based on G*Power 3.1.9.7 software. G*Power uses Cohen’s effect size measure to determine an appropriate effect size, according to the type of test selected. This study applied multiple linear regression analysis and used the conventional values of Cohen’s effect size for the F-test (multiple regression), which was 0.15. There were 14 independent variables, an effect size of 0.15, an alpha error of 0.05, and a power of 0.95 [31]. 

We designed an online questionnaire with the Google Docs survey program and uploaded the URL of the questionnaire on the university website. We set the participation limit at 241 (in anticipation of drop-out rates) between 28 and 29 April 2020. The first part of the questionnaire contained instructions and an explanation of the study. It was clearly specified that personal identifying information would not be collected. Ultimately, 239 valid questionnaires were collected. The participation of each student was completely voluntary, and they could withdraw at any stage of the study. 

### 2.3. Measurements

#### 2.3.1. Preventive Behaviors Related to COVID-19

Preventive behaviors refer to the preventive actions related to the spread of COVID-19. In this study, preventive behaviors were measured with 10 items addressing hand hygiene (frequency and methods), mobile phone hygiene, wearing masks (frequency, method, and not touching the surface), coughing etiquette, social distancing, and infodemic behaviors (producing and sharing). Questions were rated on a 5-point Likert-type scale ranging from 1 (“not at all”) to 5 (“all the time”). Higher scores reflected good preventive behavior. 

#### 2.3.2. Perceived Susceptibility

Perceived susceptibility refers to the perception of being at risk of developing a certain disease [16]. In this study, two items were used to measure the risk perception of susceptibility to COVID-19 on a 5-point Likert-type scale: “The COVID-19 pandemic is an issue that is highly relatable to me.” and “I am more susceptible to COVID-19 infection.” The total score ranged from 2 to 10 points, and a higher score indicated higher perceived susceptibility to COVID-19. A previous study showed a Cronbach’s alpha coefficient of 0.58 [32], and the Cronbach’s alpha in this study was 0.53.

#### 2.3.3. Perceived Severity

Perceived severity refers to the degree to which an individual is seriously aware of the disease and to what extent it affects their life [16]. In this study, three items were measured to determine the risk perception of the severity of COVID-19 on a 5-point Likert-type scale: “Being infected with COVID-19 will affect my studies, daily life, and family negatively.” The total score ranged from 3 to 15 points, and a higher score indicated higher perception of the severity of COVID-19. A previous study showed a Cronbach’s alpha coefficient of 0.63 [32], and the Cronbach’s alpha in this study was 0.57.

#### 2.3.4. Issue Involvement

Issue involvement was measured with three items addressing importance, interest, and relevance on a 5-point Likert-type scale: “I consider the COVID-19 pandemic as an important issue.”; “The COVID-19 pandemic is an issue that draws attention.”; and “The COVID-19 pandemic is an issue that is highly relatable to me.” The total score ranged from 3 to 15 points, and a higher score indicated higher issue involvement. A previous study showed a Cronbach’s alpha coefficient of 0.88 [29], while the Cronbach’s alpha in this study was 0.79. 

#### 2.3.5. Subjective Norms

Subjective norms were measured using 10 items, including 5 items related to normative beliefs and 5 items corresponding to motivations, and rated on a 5-point Likert-type scale. The score was calculated according to the criteria of the item development recommendation of the theory of planned behavior [28]. Higher scores indicated emerging adults’ perception that parents, friends, university, and official and non-official sources believed that they should perform preventive behavior. A previous study showed a Cronbach’s alpha coefficient of 0.96 [19]. The Cronbach’s alpha in this study was 0.76. 

### 2.4. Statistical Analysis

Data analysis was conducted using the IBM SPSS 25.0 program (IBM Corporation, Armonk, NY, USA). Descriptive statistics (means, standard deviation, and proportions), an independent t-test, and analysis of variance (ANOVA) tests were used to describe general characteristics, risk perception, issue involvement, subjective norms, and preventive behaviors related to participants’ COVID-19 perceptions. Pearson’s correlation coefficients were calculated for the preliminary examination of the associations between variables. Normal distributions are usually evaluated using plots and means, medians, skewness, and kurtosis. We evaluated skewness and kurtosis to assess the normality assumption.

A hierarchical multiple regression analysis was performed with preventive behavior scores as the dependent variable. Hierarchical multiple regression is a form of multiple regression in which the variables are entered into regression based on a predetermined logical scheme. In this analysis, three hierarchical or sequential multivariate regression models were used to examine the factors associated with preventive behaviors related to COVID-19. In the first regression model (Model 1), demographic factors (sex, age) were entered; following this, the factors of risk perception and issue involvement were entered into the second regression model (Model 2). Risk perception included perceived susceptibility and perceived severity. Further, the factors of subjective norms of friends, school, parents, and official and non-official sources were entered into the final regression model (Model 3). 

### 2.5. Ethical Considerations

Instructions and an explanation of the study were provided online to the participants prior to completing the questionnaire, and no personal information that would make the participants identifiable was collected. The participants provided written informed consent to participate in the research, and those who completed the survey with valid answers were given a reward of US $2.50. This study was approved (202004-HR-1846-02) by the Research Ethics Committee of the authors’ affiliated university.

## 3. Results

### 3.1. Participant Characteristics

The general characteristics of the 239 study participants are presented in Table 1. The participants who completed the study had a mean age of 21.97 years, and 63.6% were female. Most participants were younger than 25 years and had no previous experience with infectious diseases (Table 1). The female participants generally had higher preventive behavior scores than did the males (Supplementary Table S1). 

### 3.2. Mean and SD of Study Variables

The means, SDs, skewness, and kurtosis of preventive behaviors, risk perception, issue involvement, and subjective norms are shown in Table 2. The average score for 10 preventive behaviors related to COVID-19 was 4.13/5 points. Considering the details of preventive action, the mean score for face mask usage was the highest, and the score for mobile phone hygiene was the lowest. In hand hygiene, the mean score for hand washing frequency was higher (4.64; ±0.664) than that of following handwashing methods (3.81 ± 1.014). The mean scores for coughing etiquette and infodemic were higher than the average score of 10 preventive behaviors. However, the score for social distancing was lower than the average score. The mean score for perceived susceptibility was lower than perceived severity and 4.35 for issue involvement. The highest score for subjective norms came from the parents, followed by official sources, school, friends, and non-official sources. The data used in this study satisfied the condition of normality after evaluating skewness and kurtosis [33].

### 3.3. Correlation of the Study Variables

Correlation analysis confirmed a statistically significant relationship between preventive behaviors and all four dimensions of variables. Preventive behaviors related to COVID-19 showed positive, significant correlations with subjective norms: parents (r = 0.411, *p* < 0.001), issue involvement (r = 0.249, *p* < 0.001), perceived severity (r = 0.133, *p* < 0.05), and schools (r = 0.163, *p* < 0.05). The correlations among all the study variables are presented in Table 3.

### 3.4. Factors Associated with Preventive Behaviors Related to COVID-19

The factors related to preventive behaviors among emerging adults during the pandemic were determined as follows: Three separate hierarchical regression analyses were conducted to determine how much of the variance in preventive behaviors could be explained by issue involvement, risk perception, and subjective norms after controlling for demographic characteristics. 

The Durbin–Watson value was 1.96, indicating that the residuals for these data were uncorrelated. In our study, the variance inflation (VIF) was 1.08–1.78, and the tolerance limits were 0.1 or more and 10 or less. There was no multicollinearity in the correlation between the independent variables.

In Model 1, sex (β = 0.109, *p* < 0.001) was shown to be an important factor in preventive behaviors, with an explanatory power of 1.4% (F = 2.670, *p* = 0.071). In Model 2, issue involvement (β = 0.218, *p* = 0.003) was identified as an important factor, with an explanatory power of 6.4%. In Model 3, subjective norms related to parents (β = 0.425, *p* < 0.001), issue involvement (β = 0.160, *p* = 0.024), and sex (β = 0.137, *p* = 0.029) were identified as important factors. The model fit was significant (F = 6.997, *p* < 0.001), and the explanatory power of the entire model was 20.1% (Table 4).

## 4. Discussion

### 4.1. COVID-19 Preventive Behaviors Among Emerging Adults

This study is significant in that it reflects the immediate response of emerging adults in Korea to a pandemic, as the study was conducted during a severe period of the pandemic. 

The study shows an increase in self-reported preventive behavior compared to the 2009 H1N1 pandemic [34] and the 2015 Middle East Respiratory Syndrome (MERS) outbreak in Korea [35]. These scores are possibly the result of intensive nationwide education and clear guideline issuance after the lessons from the MERS outbreak were learned. The national and local governments’ efforts to keep citizens informed during the COVID-19 pandemic through official government briefings to the media twice a day, sending mobile alerts, and providing guidelines to college students at all schools can be said to have brought this positive change [1].

The study shows the potential disparity between the preventive behavior score and the quality of the methods themselves. Even though the scores for preventive behaviors against COVID-19 such as coughing etiquette, hand washing frequency, and infodemic prevention were high, the detailed qualitative scores for “washing for more than 30 s with water and soap”; “maintaining the covering on the mouth and nose and not lowering the mask to the chin”; and “not touching the outer surface of the mask” were below average. Although wearing masks is an essential preventive measure [36], the effect is not guaranteed if the mask is not worn properly [30]. Therefore, the awareness of correct preventive methods is essential among young adults.

This study is also significant because infodemic behavior was included as a preventive behavior. The COVID-19 outbreak in 2019–2020 has led not only to a global public health emergency but also to an infodemic [37]. Previous research found that people who kept up to date with information during the MERS outbreak in 2015 displayed a lot of information sharing behavior [38]. In this study, only 3% of the participants shared unverified COVID-19-related materials or videos with their friends or on social network services. Any emerging adult familiar with social media and communication technologies could become a producer or a user of an infodemic in the current digital era. Infodemics arise during crises and are hard to control [39]. In the future, preventive measures should also include an infodemic control plan.

The necessity of mobile phone hygiene has emerged due to the COVID-19 pandemic, and there is a requirement for education about the risk of infection through personal items such as mobile phones [4,40].

In addition, adherence to social distancing showed low scores in this study, which could potentially be explained by the general behavior of emerging adults. As emerging adulthood is the healthiest period in an individual’s life span and a period when social networks gradually expand [41], it is possible that social distancing is more difficult for emerging adults due to their requirement for social interactions and networking. Social distancing is one of the most efficient methods of preventing the spread of the virus [5]. Therefore, the present study provides a new focus point for future studies to expand the literature on preventive measures. Effective ways for emerging adults to socially engage without compromising on social distancing could be identified. It is thus important for future studies to further develop preventive measures to help maintain social relationships while maintaining physical distance [42].

### 4.2. Factors Related to Preventive Behaviors against COVID-19 among Emerging Adults

The main finding of this study was that sex, issue involvement, and subjective norms related to parents were identified as important factors of COVID-19 preventive behaviors among emerging adults, while risk perception was not.

Female participants showed higher scores than male participants, and sex was the factor most significantly related to COVID-19 preventive behaviors in this study. This result is consistent with previous research [2], which has found that women are more compliant with preventive rules than men. Men may require further education to comply with preventive rules and to understand the importance of establishing good health and preventive behaviors during emerging adulthood.

Issue involvement is a factor that has frequently been used to describe health-related behaviors [23,43] and is identified as one of the important factors related to preventive behaviors in this study. More exposure to information may lead to action [38]. The present results may suggest the requirement for a strategy to increase emerging adults’ level of engagement in issues such as health behaviors.

This finding also emphasizes the importance of the parents’ role in educating and monitoring young adults’ compliance with COVID-19 preventive behaviors, according to the results of emerging adults’ response to the subjective norms of parents. Emerging adulthood may be a particularly important time for intervening and establishing long-term health behavior patterns, which can affect families and society because of the close relationships at this time [25]. Appropriate mediation by parents may help improve preventive behavior among emerging adults.

However, contrary to the results of previous studies, the present study found no correlation between risk perception and preventive behaviors. This result may be attributed to the use of a tool that was not previously validated—the alpha levels, measuring the internal consistency of variables of risk perception, were below 0.60. These are inadequate and suggest that the measure lacks consistency to form a single main concept. It is a major limitation of this study. Therefore, additional research is required to identify the effects of risk perception among emerging adults on COVID-19 preventive behaviors, using more reliable measures.

There are three other limitations. The study used a self-reported questionnaire that was not previously validated, except in terms of content, by experts. Therefore, it is necessary to be cautious in interpreting the results.

Second, the participants were selected from only one university, making the study confined to young, well-educated students who had access to the university website, affecting the generalizability of the results. Third, the results did not infer any causality.

Despite its limitations, the study adds to our understanding of the various characteristics of COVID-19 preventive behaviors among emerging adults because it was conducted during the peak of the pandemic in South Korea.

## 5. Conclusions

The empirical findings of this study provide a new understanding of the various characteristics and immediate responses of preventive behaviors among emerging adults during the peak of the COVID-19 pandemic in South Korea. The findings of this study suggest the roles of sex (female), issue involvement, and subjective norms of parents in promoting preventive behaviors of COVID-19. This study also contributes to research on the infodemic, which is rampant during health crises and is difficult to control. It has gained greater importance with the prevalence of ICTs. This study highlights the importance of mobile phone hygiene in relation to the risk of infection, which requires further research in the future.

## Figures and Tables

**Table 1:** General characteristics and COVID-19 preventive behavior (*N* = 239).

Variables: Mean (±SD)	Categories	*n* (%)
Sex	Male	87 (36.4)
	Female	152 (63.6)
Age: 21.97 (±2.48)	<22 years	102 (42.7)
	22–26 years	116 (48.5)
	≥26 years	21 (8.8)
Self-rated health	Poor	12 (5.0)
	Moderate	35 (14.6)
	Healthy	132 (55.2)
	Very Healthy	60 (25.1)
Novel infectious disease experience	Yes	32 (13.4)
	No	207 (86.6)

Note: SD: standard deviation.

**Table 2:** Measurement of the study variables and reliability (*N* = 239).

Dimension of Variables	Variables	Number of Items	Mean (±SD)	Skewness	Kurtosis	Cronbach’s α
Preventive behaviors	Hand hygiene	2	4.64 (±0.664)	−0.95	0.95	0.521
			3.81 (±1.014)			
	Mobile phone hygiene	1	3.07 (±1.336)	−0.06	−1.20	
	Mask	3	4.74 (±0.681)	−0.93	1.30	
			4.36 (±0.942)			
			3.21 (±1.156)			
	Coughing etiquette	1	4.41 (±0.898)	−0.16	1.90	
	Social distancing	1	3.80 (±1.101)	−0.78	−0.13	
	Infodemic	2	4.72 (±0.870)	−0.27	7.00	
			4.58 (±0.885)			
Risk perception	Perceived susceptibility	2	2.67 (±0.832)	−0.15	−0.63	0.531
	Perceived severity	3	4.65 (±0.510)	−2.30	6.90	0.573
Issue involvement	Issue involvement	3	4.35 (±0.682)	−0.86	0.17	0.792
Subjective norm	Friend	2	11.64 (±5.504)	0.47	0.05	0.755
	Parents	2	17.49 (±4.982)	−0.43	−0.04	
	School	2	14.82 (±5.143)	0.19	−0.37	
	Non-official sources	2	11.45 (±5.341)	0.32	−0.19	
	Official sources	2	15.79(±5.013)	−0.01	−0.35	

Range of preventive behaviors, risk perception, issue involvement: 1–5; subjective norms: 1–25; SD: standard deviation.

**Table 3:** Correlations between the study variables.

	1	2	3	4	5	6	7	8	9
1. Preventive behaviors	1								
2. Perceived susceptibility	0.167 **	1							
3. Perceived severity	0.133 *	0.260 **	1						
4. Issue involvement	0.249 **	0.393 **	0.359 **	1					
5. Subjective norm (friends)	0.023	0.083	0.106	0.169**	1				
6. Subjective norm (parents)	0.411 **	0.115	0.179 **	0.295 **	0.299 **	1			
7. Subjective norm (school)	0.163 *	0.072	0.189 **	0.247 **	0.338 **	0.489 **	1		
8. Subjective norm (non-official sources)	0.059	0.152 *	0.161 *	0.244 **	0.484 **	0.212 **	0.461 **	1	
9. Subjective norm (official sources)	0.070	0.117	0.319 **	0.385 **	0.229 **	0.310 **	0.488 **	0.507 **	1

Note: ** = *p* < 0.001, * = *p* < 0.05.

**Table 4:** Factors related to COVID-19 preventive behaviors among emerging adults.

Variables	Model 1			Model 2			Model 3		
	β	t	*p*	β	t	*p*	β	t	*p*
Sex	0.109	1.62	<0.001	0.101	1.519	0.130	0.137	2.192	0.029
Age	−0.075	−1.12	0.107	−0.0053	−0.801	0.424	−0.004	−0.066	0.947
Perceived susceptibility				0.049	0.697	0.487	0.046	0.704	0.482
Perceived severity				0.021	0.312	0.755	0.018	0.270	0.787
Issue involvement				0.218	3.041	0.003	0.160	2.270	0.024
Subjective norm (friends)							−0.134	−1.929	0.055
Subjective norm (parents)							0.425	6.131	<0.001
Subjective norm (school)							0.014	0.176	0.860
Subjective norm (non-official sources)							0.048	0.614	0.540
Subjective norm (official sources)							−0.145	−1.879	0.061
*F* (*p*)	2.670 (0.071)			4.270 (0.001)			6.997 (<0.001)		
R^2^	0.022			0.084			0.235		
Adjusted R^2^	0.014			0.064			0.201		

Hierarchical regression analyses.

## Data Availability

The data presented in this study are available on request from the corresponding author. The data are not publicly available due to privacy.

## Supplementary Materials

The following are available online at https://www.mdpi.com/1660-4601/18/5/2569/s1, Table S1: Sex difference in preventive behaviors related to COVID-19.
