# Supplementary material for: Preventive Behavioral Insights for Emerging Adults: A Survey during the COVID-19 Pandemic
Source: Int J Environ Res Public Health. 2021 Mar 4;18(5):2569. doi: 10.3390/ijerph18052569 (PMC7967644; doi:10.3390/ijerph18052569)
Supplement: Supplementary file 1 [file ijerph-18-02569-s001.pdf]

Table S1. Sex difference in preventive behaviors related to COVID-19.

ITEM		Not at all	rarely	Some times	Freque ntly	All the time
Hand hygiene						
1	I wash my hands immediately upon returning home					
2	I wash my hands for at least 30 seconds with soap and water					
Mobile phone hygiene						
3	I disinfect my phone or other objects before using for sanitation					
Mask						
4	I always wear a face mask when going outside					
5	When I wear a mask, I keep my nose and mouth covered at all times					
6	I do not touch the outside of my mask					
Coughing						
7	When I sneeze or cough, I do not use my hand to cover my mouth but use a tissue or my sleeve					
Social distancing behaviors						
8	I have postponed or cancelled gatherings to prevent COVID-19 infection					
Infodemic						
9	I have made unverified COVID-19 related materials or videos					
10	I have shared unverified COVID-19 related materials or videos to my friends or on SNS					
Perceived susceptibility						
1	The COVID-19 pandemic is an issue that is highly relatable to me					
2	I am more susceptible to COVID-19 infection					
Perceived severity						
3	Being infected with COVID-19 will affect my studies					
4	Being infected with COVID-19 will affect my daily life					
5	Being infected with COVID-19 will affect my family negatively					
Issue involvement						
6	I consider the COVID-19 pandemic as an important issue					
7	The COVID-19 pandemic is an issue that draws attention					
8	The COVID-19 pandemic is an issue that is highly relatable to me					
Subjective Norms						
9	11. My friends encourage me to follow the COVID-19 prevention guidelines					
10	12. My parents encourage me to follow the COVID-19 prevention guidelines					
11	My school and teachers encourage me to follow the COVID-19 prevention guidelines					
12	YouTube and SNS encourage me to follow the COVID-19 prevention guidelines					
13	The news, both print and online, encourage me to follow the COVID-19 prevention guidelines					

14	I tend to follow my friends' advice					
15	I tend to follow my parents' advice					
16	I tend to follow advice given by my school and teachers					
17	I tend to follow advice given by YouTube and SNS					
18	I tend to follow advice given by the news, both print and offline					
